# Triglyceride-glucose index as a suitable non-insulin-based insulin resistance marker to predict cardiovascular events in patients undergoing complex coronary artery intervention: a large-scale cohort study

**DOI:** 10.1186/s12933-023-02110-0

**Published:** 2024-01-06

**Authors:** Jining He, Chenxi Song, Sheng Yuan, Xiaohui Bian, Zhangyu Lin, Min Yang, Kefei Dou

**Affiliations:** 1 State Key Laboratory of Cardiovascular Disease, Beijing, China; 2 Cardiometabolic Medicine Center, Fuwai Hospital, National Center for Cardiovascular Diseases, Chinese Academy of Medical Sciences and Peking Union Medical College, Beijing, China; 3 Department of Cardiology, Fuwai Hospital, National Center for Cardiovascular Diseases, Chinese Academy of Medical Sciences and Peking Union Medical College, A 167 Beilishi Rd, Xicheng District, Beijing, 100037 China; 4 National Clinical Research Center for Cardiovascular Diseases, Beijing, China

**Keywords:** Coronary artery disease, Clinical relevance, Insulin resistance, Percutaneous coronary intervention, Prognosis

## Abstract

**Background:**

Insulin resistance (IR), a hallmark of proceeding diabetes and cardiovascular (CV) disease, has been shown to predict prognosis in patients undergoing percutaneous coronary intervention (PCI). The triglyceride-glucose (TyG) index, triglyceride to high-density lipoprotein cholesterol (TG/HDL-C) ratio and metabolic score for insulin resistance (METS-IR) have been shown to be simple and reliable non-insulin-based surrogates for IR. However, limited studies have determined the associations between distinct non-insulin-based IR markers and CV outcomes in patients undergoing complex PCI who are at higher risk of CV events after PCI. Therefore, this study aimed to investigate and compare the prognostic value of these markers in patients undergoing complex PCI.

**Methods:**

This was a descriptive cohort study. From January 2017 to December 2018, a total of 9514 patients undergoing complex PCI at Fuwai Hospital were consecutively enrolled in this study. The 3 IR indices were estimated from the included patients. The primary study endpoint was CV events, defined as a composite of CV death, nonfatal myocardial infarction and nonfatal stroke.

**Results:**

During a median follow-up of 3.1 years, 324 (3.5%) CV events occurred. Multivariable Cox regression models showed per-unit increase in the TyG index (hazard ratio [HR], 1.42; 95% confidence interval [CI] 1.13–1.77), rather than per-unit elevation in either Ln(TG/HDL-C ratio) (HR, 1.18; 95%CI 0.96–1.45) or METS-IR (HR, 1.00; 95%CI 0.98–1.02), was associated with increased risk of CV events. Meanwhile, adding the TyG index to the original model led to a significant improvement in C-statistics (0.618 vs. 0.627, P < 0.001), NRI (0.12, P = 0.031) and IDI (0.14%, P = 0.003), whereas no significant improvements were observed when adding Ln (TG/HDL-C ratio) or METS-IR (both P > 0.05) to the original model.

**Conclusions:**

The TyG index, not TG/HDL-C ratio and METS-IR, was positively associated with worse CV outcomes in patients undergoing complex PCI. Our study, for the first time, demonstrated that the TyG index can serve as the suitable non-insulin-based IR marker to help in risk stratification and prognosis in this population.

**Supplementary Information:**

The online version contains supplementary material available at 10.1186/s12933-023-02110-0.

## Introduction

Complex percutaneous coronary intervention (PCI), accounting for up to 40% of the PCI procedures, was associated with worse clinical outcomes than non-complex procedures [1–3]. Previous data from a single-center PCI registry including 10,167 consecutive patients undergoing PCI suggested that the complex interventional procedures had increased hazard ratios (HRs) for major adverse cardiac events compared with non-complex PCI procedures after adjusting for confounding factors during a median follow-up of 29 months[1]. Similarly, Giustino et al. [2] showed that patients who underwent complex PCI had 1.98-fold risk of major adverse cardiac events (MACEs), indicating that alongside well-established clinical risk factors, procedural complexity is also a crucial factor for patients’ clinical management. Therefore, it is critical to identify patients undergoing complex PCI who are at a high risk of cardiovascular (CV) events so that intense strategies can be provided.

Insulin resistance (IR) is a state of reduced sensitivity and responsiveness to the action of insulin and has been shown to be a hallmark of proceeding diabetes and CV diseases (CVDs) [4, 5]. Arguably, the gold standards of IR detection are euglycemic insulin clamp and intravenous glucose tolerance testing. Nevertheless, both methods were limited in clinical application due to invasiveness and high cost [6]. Besides, the homeostasis model assessment estimated insulin resistance (HOMA-IR) index, a marker to detect β-cell function and IR, is widely used, whose practical value is greatly limited in patients receiving insulin treatment or not having functional β-cells [7]. Recently, several surrogates have been developed and proven to reliably assess IR in individuals with or without diabetes, including triglyceride-glucose (TyG) index, triglyceride to high-density lipoprotein cholesterol (TG/HDL-C) ratio, and metabolic score for insulin resistance (METS-IR) [4, 8–10]. Existing evidence have demonstrated that these 3 markers are in association with a spectrum of CV risk factors, including diabetes, metabolic syndrome, and arterial stiffness progression as well as the presence of coronary artery disease (CAD) and subsequent CV events [4, 11–13]. However, the prognostic value of these non-insulin-based IR indices in patients undergoing PCI who were at higher risk of adverse events remained unclear, and limited studies have compared these 3 markers in terms of predictive performance for CV events. Therefore, this prospective cohort study aimed to investigate the associations between the 3 non-insulin-based IR indices and CV outcomes in patients undergoing complex PCI, and sought to explore the suitable marker to predict the risk of CV events in this population.

## Methods

### Study design and population

**Fig. 1:**
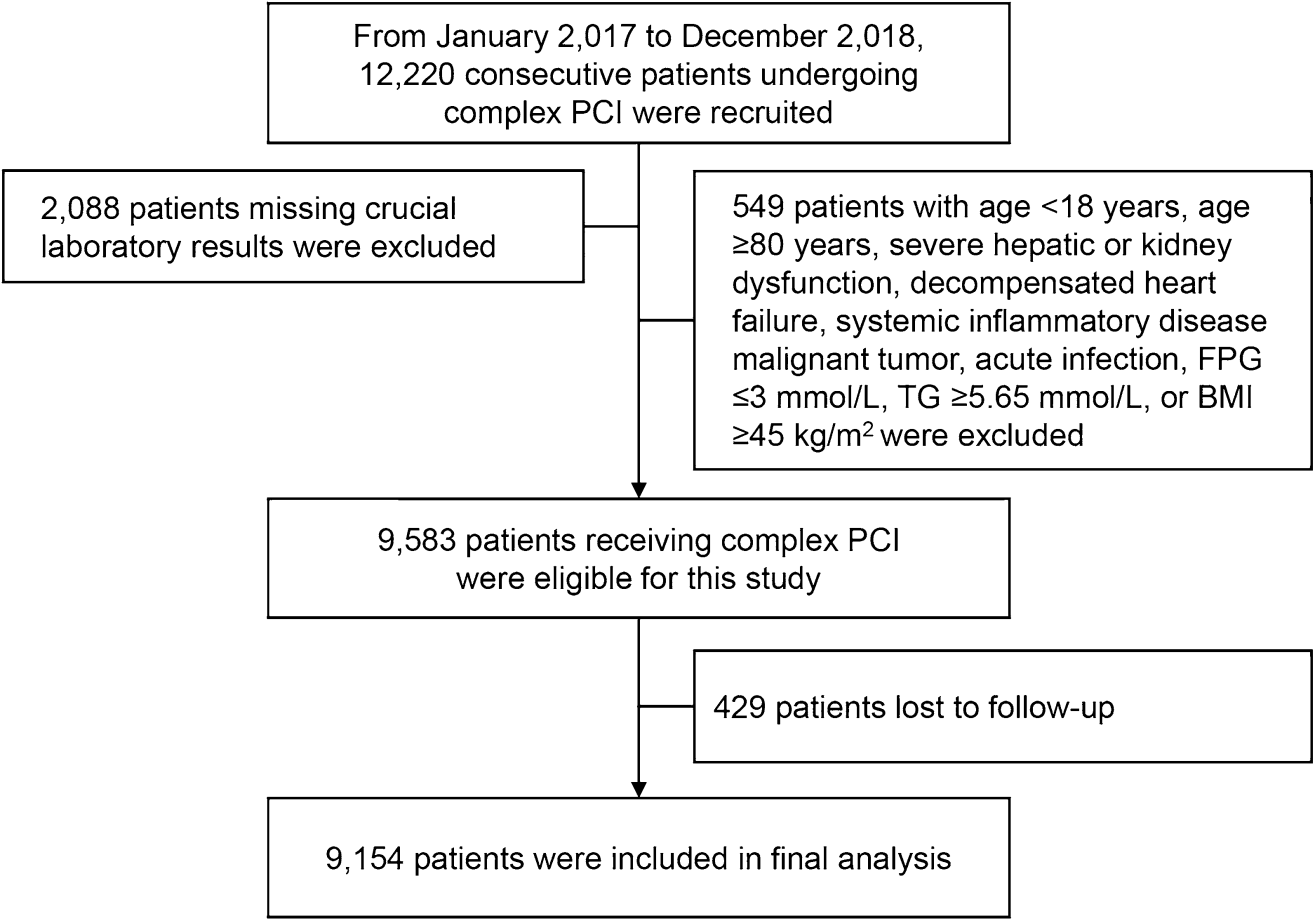
study flowchart. Complex PCI was defined as having at least 1 of the following features: 3 or more stents implanted, 3 or more lesions treated, bifurcation PCI, total stent length 60 mm or greater, left main PCI, or heavy calcification. *BMI* body mass index, *FPG* fasting plasma glucose, *PCI* percutaneous coronary intervention, *TG* triglyceride

The present study was a descriptive cohort study. From January 2017 to December 2018, 12,220 consecutive patients undergoing complex PCI were recruited from Fuwai Hospital, Chinese Academy of Medical Sciences. Complex PCI was defined as having at least 1 of the following features: 3 or more stents implanted, 3 or more lesions treated, bifurcation PCI, total stent length 60 mm or greater, left main PCI, or heavy calcification [14]. Inclusion criteria were: (1) age ≥ 18 years and < 80 years; and (2) underwent complex PCI. Patients who met the following criteria were excluded: (1) missing crucial laboratory results; (2) severe hepatic or kidney dysfunction; (3) decompensated heart failure; (4) systemic inflammatory disease; (5) malignant tumor; (6) acute infection; (7) FPG ≤ 3 mmol/L; (8) TG ≥ 5.65 mmol/L; (9) BMI ≥ 45 kg/m^2^; and (10) lost to follow-up. Finally, 9154 patients undergoing complex PCI were included in this study (Fig. 1). Baseline demographic characteristics, angiographic and procedural information, medications, and follow-up data were systematically and prospectively collected in the dedicated database by independent research personnel.

This study was conducted in compliance with the Declaration of Helsinki. The study protocol was approved by Fuwai Hospital's Institutional Review Board. All patients provided written informed consent before enrollment.

### Procedures and medication

PCI was performed by experienced interventionalists in accordance with standard techniques. The choice of devices, adjunctive examinations (i.e., intravascular ultrasound and optical coherence tomography), and detailed strategies were at operators’ discretions. Before the scheduled PCI, aspirin (300 mg) and a P2Y12 inhibitor (clopidogrel 300–600 mg or ticagrelor 180 mg) were administered to all patients unfractionated heparin or bivalirudin were used to achieve procedural anticoagulation. After the catheterization, aspirin 100 mg/day was prescribed indefinitely and clopidogrel 75 mg/day typically for 12 months. Data were entered in a dedicated database by independent research personnel [15, 16].

### Anthropometric and laboratory measurements

Anthropometric measurements, including body weight, height, and blood pressure (BP), were performed by trained study nurses according to standard protocols. BMI was estimated by dividing weight (kg) by height (m) squared. The BP measurement was performed on the non-dominant arm, using an automated electronic device (Omron model HEM-752 FUZZY; Omron Company, Dalian, China). The BP measurements were taken with participants in a seated position after 5 min of quiet rest.

On admission, venous blood samples were collected from each patient after at least 12-h fasting in the morning, and analyzed in the clinical chemistry department of Fuwai Hospital [17]. An automated biochemical analyzer (Hitachi 7150, Tokyo, Japan) was used to measure the concentrations of total cholesterol (TC), high-density lipoprotein cholesterol (HDL-C), TG, fasting plasma glucose (FPG), serum creatinine, and high sensitivity C-reactive protein (hsCRP) with an enzymatic assay. Tosoh Automated Glycohemoglobin Analyzer (HLC-723G8, Tokyo, Japan) was used to estimate glycosylated hemoglobin A1c (HbA1c). The low-density lipoprotein cholesterol (LDL-C) was determined by the Friedman equation. The eGFR was calculated by the Chinese modified MDRD equation [18].

### Follow-up, study endpoints and definitions

After the index PCI procedures, patients were followed up at 1, 6, and 12 months and annually thereafter until 3 years. Follow-up data were collected through medical records, telephone communications, or clinical visits by well-trained cardiologists who were blind to this study purpose. The median follow-up duration was 3.1 years (interquartile range [IQR]: 3.0 to 3.3 years).

The primary endpoint was defined as CV events at 3-year follow-up defined as a composite of CV death, non-fatal myocardial infarction (MI), and non-fatal stroke. The second endpoint was 3-year major adverse cardiovascular events (MACEs) defined as a composite of CV death and non-fatal MI. Unless a clear non-cardiovascular reason could be proven, all deaths were deemed CV related. According to the fourth universal definition of MI, clinical and laboratory criteria were used to determine the diagnosis. A new focal neurological deficit lasting more than 24 h that is established by neurologists using imaging data is referred to as a stroke. All events were adjudicated independently by two experienced clinicians who were blinded to the study, and any disagreements were settled by consulting a third expert.

According to American Diabetes Association criterion, diabetes was determined by previous physician diagnosis of diabetes, or FPG ≥ 126 mg/dL (7.0 mmol/L), or HbA1c levels ≥ 6.5%, or 2-h blood glucose of oral glucose tolerance test (OGTT) ≥ 200 mg/dL (11.1 mmol/L), or receiving hypoglycemic medication [19].

According to the American College of Cardiology (ACC) and American Heart Association (AHA) High Blood Pressure Clinical Practice Guideline, hypertension was defined as a systolic BP of ≥ 130 or a diastolic BP of ≥ 80 mmHg [20].

### Statistical analysis

Continuous variables are expressed as mean ± standard deviation compared by the Student’s t-test or Mann–Whitney U test, as appropriate. Categorical variables are presented as count (percentage) and compared by the Chi-square test or Fisher’s exact test, as appropriate.

The incidence of CV events and MACEs among groups was depicted using Kaplan–Meier survival curves and compared by the Log-rank test. Spearman correlation analysis was performed to estimate the correlation between 3 non-insulin-based IR indices and clinical risk factors. Restricted cubic spline (RCS) analysis adjusted for age and sex was performed to evaluate linearity assumptions of the relationship between 3 non-insulin-based IR indices and CV events. Cox proportional hazard models were adopted to investigate the association between 3 non-insulin-based IR indices and study endpoint. HRs and 95% confidence intervals (CIs) were presented**.** The multivariable Cox models were adjusted for age, male sex, BMI, acute coronary syndrome (ACS) presentations, previous myocardial infarction (MI), previous percutaneous coronary intervention (PCI), previous coronary artery bypass grafting (CABG), hypertension, diabetes, previous stroke, current smoker, LVEF, eGFR, TC, LDL-C, hsCRP, SYNTAX score, total stent number, aspirin use and statins use. To evaluate whether adding the TyG index, Ln(TG/HDL-C ratio) and METS-IR to the Age, Creatinine, and Ejection Fraction (ACEF) score [21–23], an established score for predicting adverse events after PCI, could improve the ability for predicting CV events, we calculated Harrell’s C-statistic, the continuous net reclassification improvement (NRI) and integrated discrimination improvement (IDI). A two-tailed P value < 0.05 indicated statistical significance. All statistical analyses were performed using R software version 4.1.2 (R Foundation for Statistical Computing, Vienna, Austria).

## Results

### Baseline characteristics stratified by the occurrence of CV events

The mean age of overall population was 59.83 years and 78.9% of them were males. As shown in Table 1, compared with patients not experiencing CV events, those who suffered from CV events had significantly higher TyG index levels (8.92 ± 0.56 vs. 9.02 ± 0.60, *P* = 0.002) and similar TG/HDL-C and METS-IR values (both *P* > 0.05). Besides, patients with incident CV events were older and had a higher proportion of, diabetes, ACS presentations, previous MI, prior PCI, prior CABG, higher levels of SBP, creatinine, HbA1c, FPG and hsCRP, but lower levels of BMI, LVEF and eGFR, than those without CV events (all P < 0.05). When it came to procedural information, patients in the CV events group were more likely to have a higher SYNTAX score, fewer total stent numbers, and more severely calcified lesions than those in the without CV events group (all P < 0.05). Moreover, no between-group differences were observed in terms of medications (all *P* > 0.05).

**Table 1:** Baseline characteristics stratified by the occurrence of CV events

	Overall (n = 9154)	Without CV events (n = 8830)	CV events (n = 324)	*P* value
TyG index	8.92 ± 0.56	8.92 ± 0.56	9.02 ± 0.60	0.002
TG/HDL-C ratio	3.83 ± 2.46	3.83 ± 2.46	3.94 ± 2.53	0.432
METS-IR	41.68 ± 7.02	41.69 ± 7.01	41.57 ± 7.22	0.762
Age, years	59.83 ± 9.64	59.73 ± 9.62	62.66 ± 9.88	< 0.001
Male	7220 (78.9)	6975 (79.0)	245 (75.6)	0.164
BMI, kg/m^2^	25.98 ± 3.16	26.00 ± 3.16	25.58 ± 3.09	0.019
Clinical presentation				0.002
CCS	3582 (39.1)	3482 (39.4)	100 (30.9)	
ACS	5572 (60.9)	5348 (60.6)	224 (69.1)	
Family history of CAD	1078 (11.8)	1034 (11.7)	44 (13.6)	0.348
Prior MI	2322 (25.4)	2210 (25.0)	112 (34.6)	< 0.001
Prior PCI	2042 (22.3)	1952 (22.1)	90 (27.8)	0.019
Prior CABG	302 (3.3)	282 (3.2)	20 (6.2)	0.005
Hypertension	7681 (83.9)	7397 (83.8)	284 (87.7)	0.073
Diabetes	4202 (45.9)	4033 (45.7)	169 (52.2)	0.025
Prior stroke	1261 (13.8)	1204 (13.6)	57 (17.6)	0.051
PAD	641 (7.0)	609 (6.9)	32 (9.9)	0.051
Current smoker	2880 (31.5)	2770 (31.4)	110 (34.0)	0.357
CKD	143 (1.6)	134 (1.5)	9 (2.8)	0.117
SBP, mmHg	131.33 ± 17.59	131.24 ± 17.57	133.65 ± 17.90	0.016
DBP, mmHg	77.38 ± 10.85	77.40 ± 10.83	76.91 ± 11.26	0.427
LVEF, %	61.85 ± 6.68	61.92 ± 6.58	59.78 ± 8.76	< 0.001
Laboratory tests				
Creatinine, μmol/L	83.41 ± 17.17	83.28 ± 16.88	87.02 ± 23.33	< 0.001
eGFR, mL/min/1.73m^2^	85.37 ± 17.50	85.53 ± 17.44	81.01 ± 18.40	< 0.001
HbA1c, %	6.53 ± 1.27	6.53 ± 1.26	6.78 ± 1.39	< 0.001
FPG, mmol/L	6.59 ± 2.42	6.56 ± 2.39	7.23 ± 3.12	< 0.001
TG, mmol/L	1.66 ± 0.81	1.66 ± 0.81	1.69 ± 0.82	0.468
TC, mmol/L	4.04 ± 1.05	4.04 ± 1.05	4.02 ± 1.04	0.730
HDL-C, mmol/L	1.10 ± 0.29	1.10 ± 0.29	1.09 ± 0.29	0.595
LDL-C, mmol/L	2.45 ± 0.91	2.46 ± 0.92	2.43 ± 0.88	0.681
hsCRP, mg/L	2.63 ± 3.05	2.61 ± 3.05	3.06 ± 3.21	< 0.001
Procedural data				
SYNTAX score	16.45 ± 5.51	16.43 ± 5.49	17.11 ± 5.91	0.028
Left main disease	1761 (19.2)	1688 (19.1)	73 (22.5)	0.144
Three-vessel disease	5057 (55.2)	4870 (55.2)	187 (57.7)	0.393
CTO lesion	1204 (13.2)	1159 (13.1)	45 (13.9)	0.752
Thrombotic lesion	118 (1.3)	113 (1.3)	5 (1.5)	0.871
Ostial lesion	1438 (15.7)	1377 (15.6)	61 (18.8)	0.135
Type B2/C lesion	7222 (78.9)	6963 (78.9)	259 (79.9)	0.690
Severe calcification	691 (7.5)	651 (7.4)	40 (12.3)	0.001
Number of treated lesions	1.90 ± 0.81	1.90 ± 0.81	1.88 ± 0.85	0.669
Total stent number	4.33 ± 2.91	4.35 ± 2.93	3.96 ± 2.37	0.019
Total stent length	63.69 ± 33.43	63.78 ± 33.49	61.23 ± 31.86	0.178
Medications				
Aspirin	6775 (74.0)	6541 (74.1)	234 (72.2)	0.495
Clopidogrel	7693 (84.0)	7418 (84.0)	275 (84.9)	0.733
Statins	8882 (97.0)	8568 (97.0)	314 (96.9)	1.000
ACEI/ARB	2378 (26.0)	2299 (26.0)	79 (24.4)	0.547
β blocker	8185 (89.4)	7892 (89.4)	293 (90.4)	0.607
Antidiabetic drugs	3227 (35.3)	3099 (35.1)	128 (39.5)	0.116

Values are mean ± standard deviation or n (%)

*CV* cardiovascular, *TyG* triglyceride-glucose, *TG* triglyceride, *HDL-C* high-density lipoprotein cholesterol, *METS-IR* metabolic score for insulin resistance, *BMI* body mass index, *CCS* chronic coronary syndrome, *ACS* acute coronary syndrome, *CAD* coronary artery disease, *MI* myocardial infarction, *PCI* percutaneous coronary intervention, *CABG* coronary artery bypass grafting, *PAD* peripheral artery disease, *CKD* chronic kidney disease, *SBP* systolic blood pressure, *DBP* diastolic blood pressure, *LVEF* left ventricular ejection fraction, *eGFR* estimated glomerular filtration rate, *HbA1c* hemoglobin A1c, *FPG* fasting plasma glucose, *TC* total cholesterol, *LDL-C* low-density lipoprotein cholesterol, *hsCRP* high sensitivity C-reactive protein, *SYNTAX* synergy between PCI with taxus and cardiac surgery, *CTO* chronic total occlusion, *ACEI* angiotensin-converting enzyme inhibitor, *ARB* angiotensin II receptor blocker

### Correlation between 3 non-insulin-based IR indices and clinical risk factors

Correlation analyses were performed to evaluate the correlation between the 3 non-insulin-based IR indices and clinical risk factors. As shown in Additional file 1: Table S1, the results showed that the TyG index was positively correlated with BMI, SBP, DBP, HbA1c, FPG, TC, TG, LDL-C, hsCRP and serum creatinine, and inversely correlated with age, HDL-C and eGFR (all *P* < 0.05). Besides, TG/HDL-C ratio was positively correlated with BMI, DBP, HbA1c, FPG, TC, TG, LDL-C, hsCRP and serum creatinine, and inversely correlated with age, SBP and HDL-C (all *P* < 0.05). Moreover, METS-IR was positively correlated with BMI, DBP, HbA1c, FPG, TG, LDL-C, hsCRP and serum creatinine, and inversely correlated with age, TC and HDL-C (all *P* < 0.05).

### Non-insulin-based IR indices and CV outcomes

**Fig. 2:**
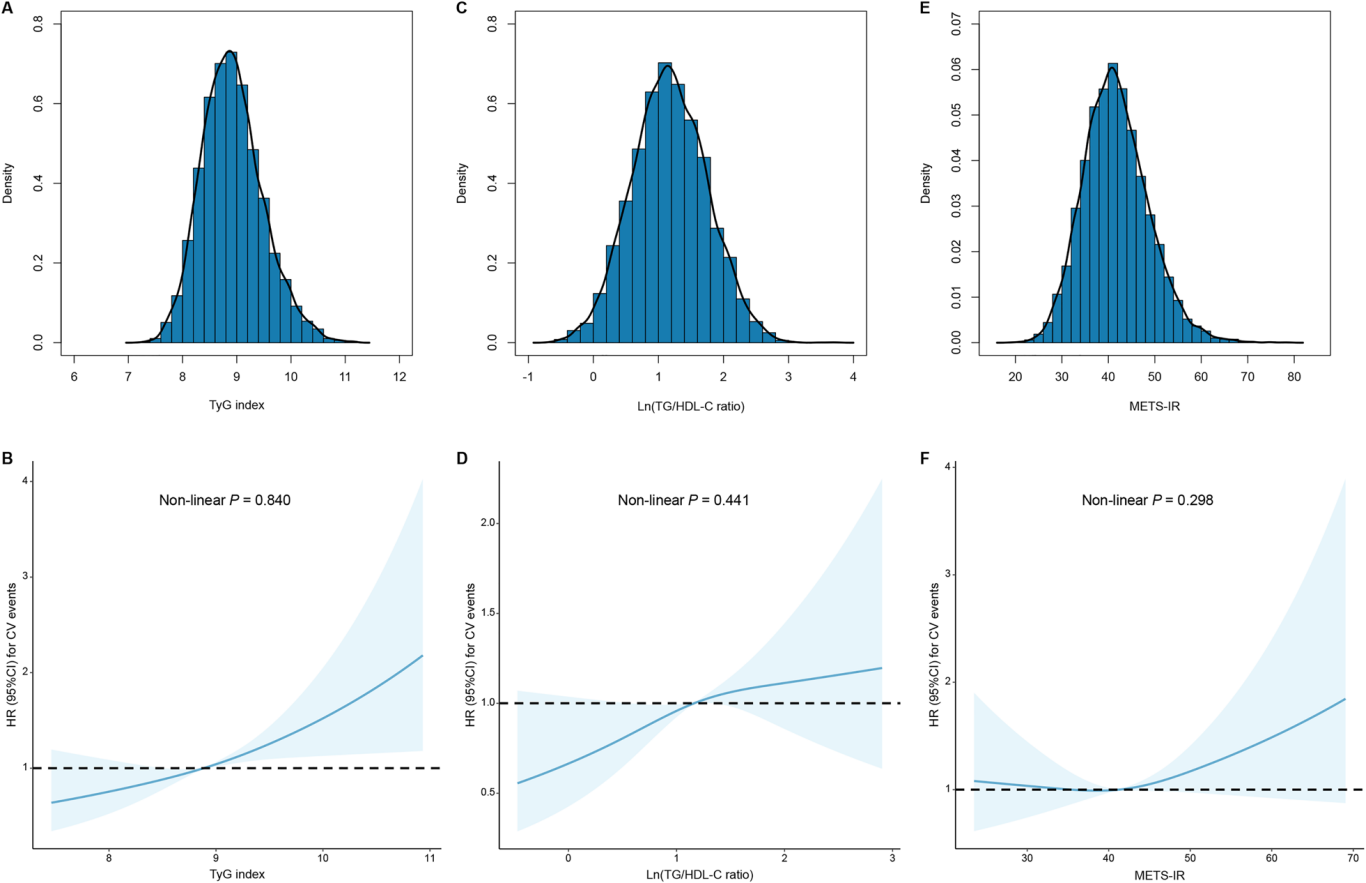
Distributions of the 3 IR indices and RCS curves for the association between the 3 IR indices and CV events. Distributions for the TyG index (**A**), TG/HDL-C ratio (**C**), and METS-IR (E); RCS curves for the TyG index (**B**), TG/HDL-C ratio (**D**), and METS-IR (**F**). CV events were defined as a composite of CV death, nonfatal MI, and nonfatal stroke. RCS analyses were performed adjusted for age and sex. *CI* confidence interval, *CV* cardiovascular, *HR* hazard ratio, *METS-IR* metabolic score for insulin resistance, *MI* myocardial infarction, *RCS* restrict cubic spine, *TG/HDL-C* triglyceride to high-density lipoprotein cholesterol, *TyG* triglyceride-glucose

During a median follow-up of 3.1 (IQR: 3.0–3.3) years, 324 (3.5%) events and 269 (2.9%) MACEs were recorded. As shown in Fig. 2, the Kaplan–Meier survival analysis showed that patients with high levels of the TyG index had significantly higher rates of CV events and MACEs compared to those with low levels of the TyG index (both log rank *P* < 0.05). Meanwhile, high levels of TG/HDL-C ratio or METS-IR did not have significantly higher rates of both CV events and MACEs (all log rank *P* > 0.05).

**Fig. 3:**
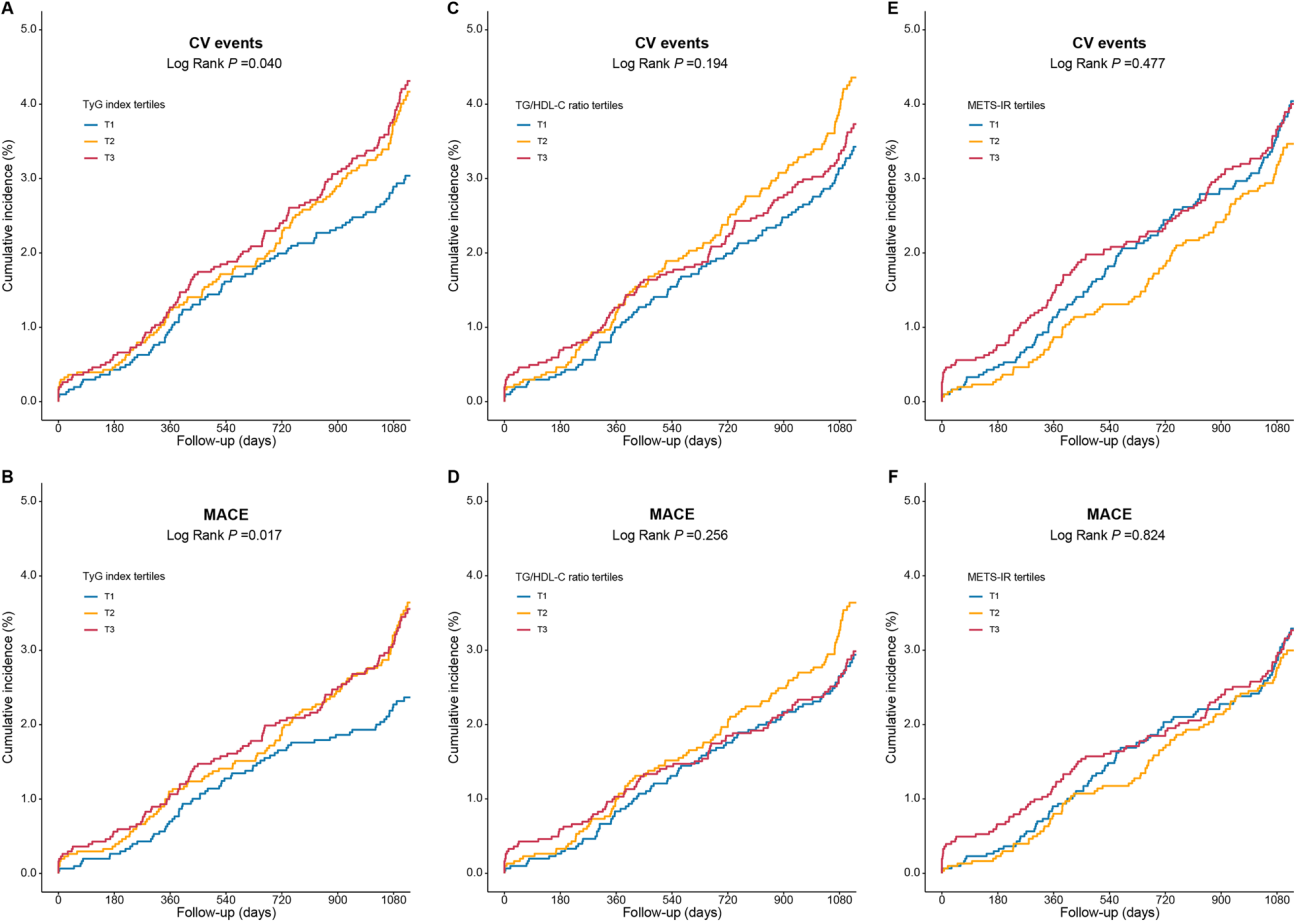
Kaplan–Meier curves according to the TyG index, TG/HDL-C ratio, and METS-IR. Kaplan–Meier curves for CV events according to the TyG index (**A**), TG/HDL-C ratio (**C**), and METS-IR (**E**); Kaplan–Meier curves for MACEs according to the TyG index (**B**), TG/HDL-C ratio (**D**), and METS-IR (**F**). CV events were defined as a composite of CV death, nonfatal MI, and nonfatal stroke. MACE was defined as a composite of CV death and nonfatal MI. *MACE* major adverse cardiac events, *CI* confidence interval, *CV* cardiovascular, *HR* hazard ratio, *METS-IR* metabolic score for insulin resistance, *MI* myocardial infarction, *RCS* restrict cubic spine, *TG/HDL-C* triglyceride to high-density lipoprotein cholesterol, *TyG* triglyceride-glucose

RCS analysis showed linear relationships between all 3 non-insulin-based IR indices and 3-year CV events after adjusted for age and male sex (Fig. 3B, D, F; all non-linear *P* > 0.05). Cox proportional hazard models were adopted to investigated the relationship between 3 non-insulin-based IR indices and CV outcomes (Table 2**, **Additional file 1: Tables S2 and S3). After multivariable adjustment, per 1-unit increase of the TyG index was associated with an increased risk of CV events and MACEs 1.41-fold and 1.39-fold, respectively, (both *P* < 0.05). In fully adjusted models, subjects in the TyG index T2 and T3 groups had higher risk of CV events compared to those in the TyG index T1 group (HR, 1.39; 95%CI 1.05–1.85; HR, 1.45; 95%CI 1.06–1.98, respectively) (Table 2). However, per 1-unit elevation in Ln(TG/HDL-C ratio) and METS-IR levels were both not associated with the risk of CV events after adjusting for confounding factors (HR, 1.03; 95%CI 0.99–1.08; HR, 1.00; 95%CI 0.98–1.02, respectively). In addition, neither T2 nor T3 groups of TG/HDL-C and METS-IR were associated with increased risk of CV events compared to T1 groups of the corresponding marker after multivariable adjustment (all *P* > 0.05) (Additional file 1: Tables S2 and S3). Similar associations were noticed between the 3 non-insulin-based markers and MACEs.

**Table 2:** the TyG index in relation to CV events and MACEs

	Events (%)	Univariable models		Multivariable models*	
		HR (95%CI)	*P* value	HR (95%CI)	*P* value
CV events^a^					
Per-unit increase in TyG index	324 (3.5)	1.34 (1.11–1.61)	0.002	1.42 (1.13–1.77)	0.002
TyG index tertiles					
T1	87 (2.9)	Reference	–	Reference	–
T2	116 (3.8)	1.34 (1.02–1.77)	0.038	1.39 (1.05–1.85)	0.023
T3	121 (4.0)	1.40 (1.06–1.84)	0.018	1.45 (1.06–1.99)	0.021
MACEs^b^					
Per-unit increase in TyG index	269 (2.9)	1.36 (1.11–1.67)	0.003	1.39 (1.09–1.78)	0.008
TyG index tertiles					
T1	68 (2.2)	Reference	–	Reference	–
T2	101 (3.3)	1.50 (1.10–2.04)	0.010	1.54 (1.12–2.11)	0.008
T3	100 (3.3)	1.48 (1.09–2.01)	0.013	1.48 (1.04–2.11)	0.027

*CV* cardiovascular, *TyG* triglyceride-glucose, *TG* triglyceride, *HDL-C* high-density lipoprotein cholesterol, *METS-IR* metabolic score for insulin resistance, *BMI* body mass index, *CCS* chronic coronary syndrome, *ACS* acute coronary syndrome, *CAD* coronary artery disease, *MI* myocardial infarction, *PCI* percutaneous coronary intervention, *CABG* coronary artery bypass grafting, *PAD* peripheral artery disease, *CKD* chronic kidney disease, *SBP* systolic blood pressure, *DBP* diastolic blood pressure, *LVEF* left ventricular ejection fraction, *eGFR* estimated glomerular filtration rate, *HbA1c* hemoglobin A1c, *FPG* fasting plasma glucose, *TC* total cholesterol, *LDL-C* low-density lipoprotein cholesterol, *hsCRP* high sensitivity C-reactive protein, *SYNTAX* synergy between PCI with taxus and cardiac surgery, *CTO* chronic total occlusion, *ACEI* angiotensin-converting enzyme inhibitor, *ARB* angiotensin II receptor blocker

^*^Models adjusted for age, male sex, BMI, ACS presentations, previous MI, previous PCI, previous CABG, hypertension, diabetes, previous stroke, current smoker, LVEF, eGFR, TC, LDL-C, hsCRP, SYNTAX score, total stent number, aspirin use and statins use

^a^CV events were defined as a composite of CV death, nonfatal MI and nonfatal stroke

^b^MACEs were defined as a composite of CV death and nonfatal MI

Finally, we assessed whether the addition of the TyG index, TG/HDL-C ratio and METS-IR to the established risk score improves risk stratification for adverse clinical events. As shown in Table 3, the C-statistic value of the ACEF score model, which predicts adverse events after PCI, was 0.618 (95% CI 0.586–0.650). The addition of the TyG index as a continuous variable to the model showed a significant improvement in C-statistic to 0.627 (95% CI 0.595–0.658, P < 0.001) and also a significant increase in NRI (0.12, *P* = 0.031) and IDI (0.14%, *P* = 0.003). However, adding Ln(TG/HDL-C ratio) and METS-IR to the original model led to numerical not significant improvement in C-statistics to 0.624 (95% CI 0.593–0.655, P = 0.061) and 0.621 (95% CI 0.589–0.652, P = 0.416), respectively. Similar results were observed regarding the improvement in MACE prediction.

**Table 3:** C-statistics of the TyG index, TG/HDL-C ratio and METS-IR for predicting CV events and MACEs in patients undergoing complex PCI

	C-statistics	*P* value	NRI	*P* value	IDI	*P* value
CV events^a^						
Original model*	0.618 (0.586–0.650)	–	Reference	–	Reference	–
Original model + TyG index	0.627 (0.595–0.658)	< 0.001	0.12	0.031	0.14%	0.003
Original model + Ln(TG/HDL-C ratio)	0.624 (0.593–0.655)	0.061	0.02	0.746	0.06%	0.044
Original model + METS-IR	0.621 (0.589–0.652)	0.416	0.06	0.281	0.01%	0.514
MACEs^b^						
Original model	0.624 (0.589–0.660)	–	Reference	–	Reference	–
Original model + TyG index	0.634 (0.599–0.668)	0.001	0.14	0.021	0.13%	0.005
Original model + Ln(TG/HDL-C ratio)	0.628 (0.593–0.663)	0.161	0.02	0.799	0.04%	0.074
Original model + METS-IR	0.628 (0.593–0.663)	0.325	0.05	0.391	0.04%	0.426

*CV* cardiovascular, *TyG* triglyceride-glucose, *TG* triglyceride, *HDL-C* high-density lipoprotein cholesterol, *METS-IR* metabolic score for insulin resistance, *BMI* body mass index, *CCS* chronic coronary syndrome, *ACS* acute coronary syndrome, *CAD* coronary artery disease, *MI* myocardial infarction, *PCI* percutaneous coronary intervention, *CABG* coronary artery bypass grafting, *PAD* peripheral artery disease, *CKD* chronic kidney disease, *SBP* systolic blood pressure, *DBP* diastolic blood pressure, *LVEF* left ventricular ejection fraction, *eGFR* estimated glomerular filtration rate, *HbA1c* hemoglobin A1c, *FPG* fasting plasma glucose, *TC* total cholesterol, *LDL-C* low-density lipoprotein cholesterol, *hsCRP* high sensitivity C-reactive protein, *SYNTAX* synergy between PCI with taxus and cardiac surgery, *CTO* chronic total occlusion, *ACEI* angiotensin-converting enzyme inhibitor, *ARB* angiotensin II receptor blocker

^*^Original model referred to the ACEF score, which was calculated by age (years)/left ventricular ejection fraction (%) + 1 (if serum creatinine value was > 2 mg/dL)

^a^CV events were defined as a composite of CV death, nonfatal MI and nonfatal stroke

^b^MACEs were defined as a composite of CV death and nonfatal MI

## Discussion

This large-scale prospective cohort study included 9154 patients undergoing complex PCI and explored the suitable non-insulin-based IR indices for predicting CV outcomes at 3-year follow-up. Salient findings are as follow: (1) multivariable Cox proportional hazard models suggested that the TyG index but not TG/HDL-C ratio or METS-IR was associated with higher risk of CV events and MACEs at 3-year follow-up; and (2) adding the TyG index to ACEF score could significantly enhanced the predictive ability for adverse clinical events, whereas no significant improvements were observed when adding TG/HDL-C ratio or METS-IR; Our study, with a sizeable sample size, firstly demonstrated that high TyG index levels, not high TG/HDL-C ratio or METS-IR levels, were associated with CV events, hard endpoints, in patients undergoing complex PCI, suggesting that the TyG index could serve as a suitable non-insulin-based IR marker for risk stratification and prognosis in this population.

Cumulative evidence has demonstrated that non-insulin-based IR indices are associated with increased coronary lesion severity and worse CV outcomes [4]. Previous studies by Mao et al. [24] and Wang et al. [25] have shown that the TyG index was positively associated the SYNTAX score (OR, 6.055; 95%CI 2.915–12.579) and the incidence of multi-vessel lesions (OR, 1.355; 95%CI 1.154–1.591), respectively. In addition, among CTO lesions, the TyG index has been shown to correlate with the occurrence of impaired collateralization and be associated with higher risk of 3-year MACCEs [11, 26]. Consistent with previous studies, our findings illustrated that higher TyG index was related to worse CV outcomes in patients undergoing complex PCI. Apart from the TyG index, existing research has demonstrated that the TG/HDL-C ratio was also a dependable marker for assessing CAD severity [9, 27]. A study by Wu et al. [9] showed that the TG/HDL-C ratio was independent predictor for CAD presence (odds ratio [OR], 1.32; 95%CI 1.02–1.70) after multivariable adjustments. Besides, Zhang et al. [27] showed that per 1-unit increase of the TG/HDL-C ratio had increased odds of multi-vessel CAD. Moreover, data from an optical coherence tomography study suggested that the TG/HDL-C ratio was related to the degree of coronary stenotic lesions and was effective in determining in-stent stenosis [28]. For prognosis, a previous study by Wang et al. [29] enrolling 2080 statins-treated CAD patients with diabetes demonstrated that TG/HDL-C ratio was also independently associated all-cause death and CV death during 4-year follow-up. Similarly, another cohort study by Wan et al. [30] showed that the TG/HDL-C ratio was a powerful predictor of all-cause death in ACS patients undergoing PCI. However, a previous investigation by Zhang et al. [31] indicated a negative association between TG/HDL-C ratio and prognosis in patients undergoing PCI. Meanwhile, previous studies have also suggested METS-IR was correlated with the CAD lesion severity and was an independent predictor for adverse clinical events [27, 31]. Whereas previous studies have shown that the relationship between METS-IR and poor prognosis was more pronounced in female, elderly or diabetic patients [10, 31]. In this study, however, neither TG/HDL-C ratio nor METS-IR was related to CV events and MACE in patients undergoing complex PCI. Meanwhile, the associations of the 3 IR indices with CV events were consistent across different subgroups.

Currently, only a few studies have compared the association of the TyG index, TG/HDL-C ratio, and METS-IR with CV disease and subsequent CV events, and the results remain somewhat controversial [8, 9, 27, 31, 32]. A previous study indicated that the TyG index and TG/HDL-C ratio were both significantly associated with a higher risk and arterial stiffness progression in hypertensive population [32]. Likewise, an analysis of 403,335 participants’ data from the UK Biobank unveiled that increased levels of the TyG index and TG/HDL-C ratio both conferred higher risk of CV diseases after multivariable adjustment including well-established CV risk factors. Importantly, this study indicated that such associations were largely mediated by greater incidence of dyslipidemia, diabetes, and hypertension [8]. However, in a longitudinal study with small population sample of 723 individuals, the HRs for incident CV disease were significantly increased when assessed by the TG/HDL-C ratio, but not by the TyG index after adjusting for age, sex and multiple covariates [33]. A previous retrospective analysis of 485 CAD patients documented that the TyG index, TG/HDL-C ratio and METS-IR were all associated with increased risk of multi-vessel lesions. The area under the curve (AUC) of the ROC plots for the TyG index, TG/HDL-C ratio, and METS-IR were 0.673, 0.652, and 0.726, respectively (all P < 0.001), suggesting that METS-IR had the highest predictive value for CAD severity, followed by the TyG index [27]. Whereas another study including 802 consecutive patients undergoing coronary angiography for suspected CAD showed that the TG/HDL-C ratio and METS-IR were independent predictors for CAD presence, and METS-IR was the only marker to independently predict the severity of CAD, evaluated by the Ginsini score [9]. Regarding CAD prognosis, a previous study by Zhang et al. [31]. illustrated that, in the multivariable models, there were no statistically significant connections between all these 3 IR indices (the TyG index, TG/HDL-C ratio, and METS-IR) and adverse CV and cerebrovascular events in patients undergoing PCI. Meanwhile, all the 3 IR markers failed to improve the predictive performance of the original risk model for MACCEs. Further subgroup analysis revealed that the associations of METS-IR with incident MACCEs could be observed in subjects with female sex or age ≥ 60 years [31]. Inconsistent with previous findings, our studies showed that the TyG index was the only non-insulin-based IR marker to predict CV events in patients after complex PCI. Meanwhile, adding the TyG index, but not TG/HDL-C ratio or METS-IR, to the ACEF score led to a significant enhancement in predictive performance for CV events and MACEs. The differences could be attributed to the large sample size, the secondary prevention study population, the inclusion of high-risk patients of CV events, and the adoption of hard endpoints in this study. Further prospective studies with large sample size are expected to compare the survival outcomes of CAD patients with different levels of IR indices.

There are several limitations in this study. First, due to the nature of observation study design, potential confounding factors cannot be fully adjusted [34, 35]. Second, follow-up data on 3 non-insulin-based IR markers was not available, which could have clinical relevance. Third, since insulin levels were not measured in patients recruited in this study, HOMA-IR cannot be estimated. Further large-scale prospective studies with long-term follow-up are needed to confirm our findings.

## Conclusions

The TyG index, not TG/HDL-C ratio and METS-IR, was associated with CV outcomes in patients undergoing complex PCI. Our study, for the first time, demonstrated that the TyG index as a suitable non-insulin-based IR marker could help in risk stratification and prognosis in this population.

### Supplementary Information

**Additional file 1: Table S1.** Correlation between the 3 insulin resistance markers and clinical risk factors. **Table S2.** TG/HDL-C ratio in relation to CV events and MACEs. **Table S3.** METS-IR in relation to CV events and MACEs.

## Data Availability

The datasets used and/or analysed during the current study are available from the corresponding author on reasonable request.
