# Supplementary material for: Triglyceride-glucose index as a suitable non-insulin-based insulin resistance marker to predict cardiovascular events in patients undergoing complex coronary artery intervention: a large-scale cohort study
Source: Cardiovasc Diabetol. 2024 Jan 6;23:15. doi: 10.1186/s12933-023-02110-0 (PMC10771666; doi:10.1186/s12933-023-02110-0)
Supplement: Supplementary file 1 — Additional file 1: Table S1. Correlation between the 3 insulin resistance markers and clinical risk factors. Table S2. TG/HDL-C ratio in relation to CV events and MACEs. Table S3. METS-IR in relation to CV events and MACEs. [file 12933_2023_2110_MOESM1_ESM.docx]

**Triglyceride-glucose index as a suitable non-insulin-based insulin resistance marker to predict cardiovascular events in patients undergoing complex coronary artery intervention: a large-scale cohort study**

**Additional file 1**

**Contents**

[Table S1 Correlation between the 3 insulin resistance markers and clinical risk factors 2](#_Toc154578102)

[Table S2 TG/HDL-C ratio in relation to CV events and MACEs 3](#_Toc154578103)

[Table S3 METS-IR in relation to CV events and MACEs 4](#_Toc154578104)

# Table S1 Correlation between the 3 insulin resistance markers and clinical risk factors

| **Variables** | **TyG index** | | **TG/HDL-C ratio** | | **METS-IR** | |
| --- | --- | --- | --- | --- | --- | --- |
|  | **β** | ***P* value** | **β** | ***P* value** | **β** | ***P* value** |
| Age, years | -0.105 | <0.001 | -0.209 | <0.001 | -0.278 | <0.001 |
| BMI, kg/m^2^ | 0.185 | <0.001 | 0.190 | <0.001 | 0.844 | <0.001 |
| SBP, mmHg | 0.027 | 0.010 | -0.047 | <0.001 | 0.008 | 0.464 |
| DBP, mmHg | 0.049 | <0.001 | 0.028 | 0.006 | 0.106 | <0.001 |
| HbA1c, % | 0.438 | <0.001 | 0.079 | <0.001 | 0.228 | <0.001 |
| FPG, mmol/L | 0.597 | <0.001 | 0.109 | <0.001 | 0.270 | <0.001 |
| TC, mmol/L | 0.284 | <0.001 | 0.121 | <0.001 | -0.044 | <0.001 |
| TG, mmol/L | 0.817 | <0.001 | 0.880 | <0.001 | 0.421 | <0.001 |
| LDL-C, mmol/L | 0.252 | <0.001 | 0.151 | <0.001 | 0.063 | <0.001 |
| HDL-C, mmol/L | -0.257 | <0.001 | -0.555 | <0.001 | -0.604 | <0.001 |
| HSCRP, mg/L | 0.070 | <0.001 | 0.082 | <0.001 | 0.126 | <0.001 |
| Creatinine, μmol/L | 0.056 | <0.001 | 0.088 | <0.001 | 0.093 | <0.001 |
| eGFR, mL/min/1.73m^2^ | -0.064 | <0.001 | -0.016 | 0.132 | 0.026 | 0.014 |

TyG, triglyceride-glucose; TG, triglyceride; HDL-C, high-density lipoprotein cholesterol; METS-IR, metabolic score for insulin resistance; BMI, body mass index; SBP, systolic blood pressure; DBP, diastolic blood pressure; HbA1c, hemoglobin A1c; FPG, fasting plasma glucose; TC, total cholesterol; LDL-C, low-density lipoprotein cholesterol; hsCRP, high sensitivity C-reactive protein; eGFR, estimated glomerular filtration rate.

# Table S2 TG/HDL-C ratio in relation to CV events and MACEs

|  | Events (%) | Univariable models | | Multivariable models* | |
| --- | --- | --- | --- | --- | --- |
|  |  | HR (95%CI) | *P* value | HR (95%CI) | *P* value |
| **CV events** |  |  |  |  |  |
| Per-unit increase in Ln(TG/HDL-C ratio) | 324 (3.5) | 1.02 (0.98-1.06) | 0.430 | 1.18 (0.96-1.45) | 0.122 |
| TG/HDL-C ratio tertiles |  |  |  |  |  |
| T1 | 97 (3.2) | Reference | - | Reference | - |
| T2 | 122 (4.0) | 1.27 (0.97-1.66) | 0.078 | 1.33 (1.00-1.76) | 0.050 |
| T3 | 105 (3.4) | 1.09 (0.83-1.44) | 0.545 | 1.19 (0.88-1.60) | 0.256 |
| **MACEs** |  |  |  |  |  |
| Per-unit increase in Ln(TG/HDL-C ratio) | 269 (2.9) | 1.01 (0.96-1.06) | 0.749 | 1.12 (0.89-1.40) | 0.337 |
| TG/HDL-C ratio tertiles |  |  |  |  |  |
| T1 | 83 (2.7) | Reference | - | Reference | - |
| T2 | 102 (3.3) | 1.24 (0.93-1.66) | 0.145 | 1.28 (0.94-1.74) | 0.117 |
| T3 | 84 (2.8) | 1.02 (0.75-1.38) | 0.912 | 1.09 (0.79-1.51) | 0.607 |

*Models adjusted for age, male sex, BMI, hypertension, diabetes mellitus, ACS presentation, histories of MI, TC, LDL-C, hsCRP, serum creatinine, LVEF, and three-vessel disease.

Abbreviations as in Table S1.

# Table S3 METS-IR in relation to CV events and MACEs

|  | Events (%) | Univariable models | | Multivariable models* | |
| --- | --- | --- | --- | --- | --- |
|  |  | HR (95%CI) | *P* value | HR (95%CI) | *P* value |
| **CV events** |  |  |  |  |  |
| Per-unit increase in METS-IR | 324 (3.5) | 1.00 (0.98-1.01) | 0.765 | 1.00 (0.98-1.02) | 0.968 |
| METS-IR tertiles |  |  |  |  |  |
| T1 | 113 (3.7) | Reference | - | Reference | - |
| T2 | 98 (3.2) | 0.86 (0.66-1.13) | 0.293 | 0.86 (0.65-1.15) | 0.318 |
| T3 | 113 (3.7) | 1.00 (0.77-1.30) | 0.978 | 1.02 (0.76-1.37) | 0.895 |
| **MACEs** |  |  |  |  |  |
| Per-unit increase in METS-IR | 269 (2.9) | 1.00 (0.98-1.02) | 0.930 | 1.00 (0.98-1.02) | 0.940 |
| METS-IR tertiles |  |  |  |  |  |
| T1 | 92 (3.0) | Reference | - | Reference | - |
| T2 | 85 (2.8) | 0.92 (0.69-1.24) | 0.596 | 0.92 (0.67-1.25) | 0.577 |
| T3 | 92 (3.0) | 1.00 (0.75-1.34) | 0.981 | 1.01 (0.73-1.40) | 0.963 |

*Models adjusted for age, male sex, hypertension, diabetes mellitus, ACS presentation, histories of MI, TC, LDL-C, hsCRP, serum creatinine, LVEF, and three-vessel disease.

Abbreviations as in Table S1.
